# Cooper pair splitting controlled by a temperature gradient

**DOI:** 10.3762/bjnano.14.7

**Published:** 2023-01-09

**Authors:** Dmitry S Golubev, Andrei D Zaikin

**Affiliations:** 1 QTF Centre of Excellence, Department of Applied Physics, Aalto University, FI-00076 Aalto, Finland; 2 I.E. Tamm Department of Theoretical Physics, P.N. Lebedev Physical Institute, 119991 Moscow, Russia; 3 National Research University Higher School of Economics, 101000 Moscow, Russia

**Keywords:** Cooper pair splitting, entanglement, quantum shot noise, superconducting hybrid nanostructures

## Abstract

Electrons in two different normal metallic electrodes attached to a sufficiently thin superconducting island may become entangled due to the effect of Cooper pair splitting. This phenomenon is of fundamental importance and may also have serious implications for developing quantum communication technologies. One way to identify Cooper pair splitting is to analyze long-range cross correlations of fluctuating currents in three-terminal hybrid normal–superconducting–normal nanostructures. Here, we theoretically investigate non-trivial behavior of cross-correlated non-local shot noise in the presence of a temperature gradient. We suggest that applying a temperature gradient may serve as an extra tool to control the phenomenon of Cooper pair splitting.

## Introduction

**Figure 1:**
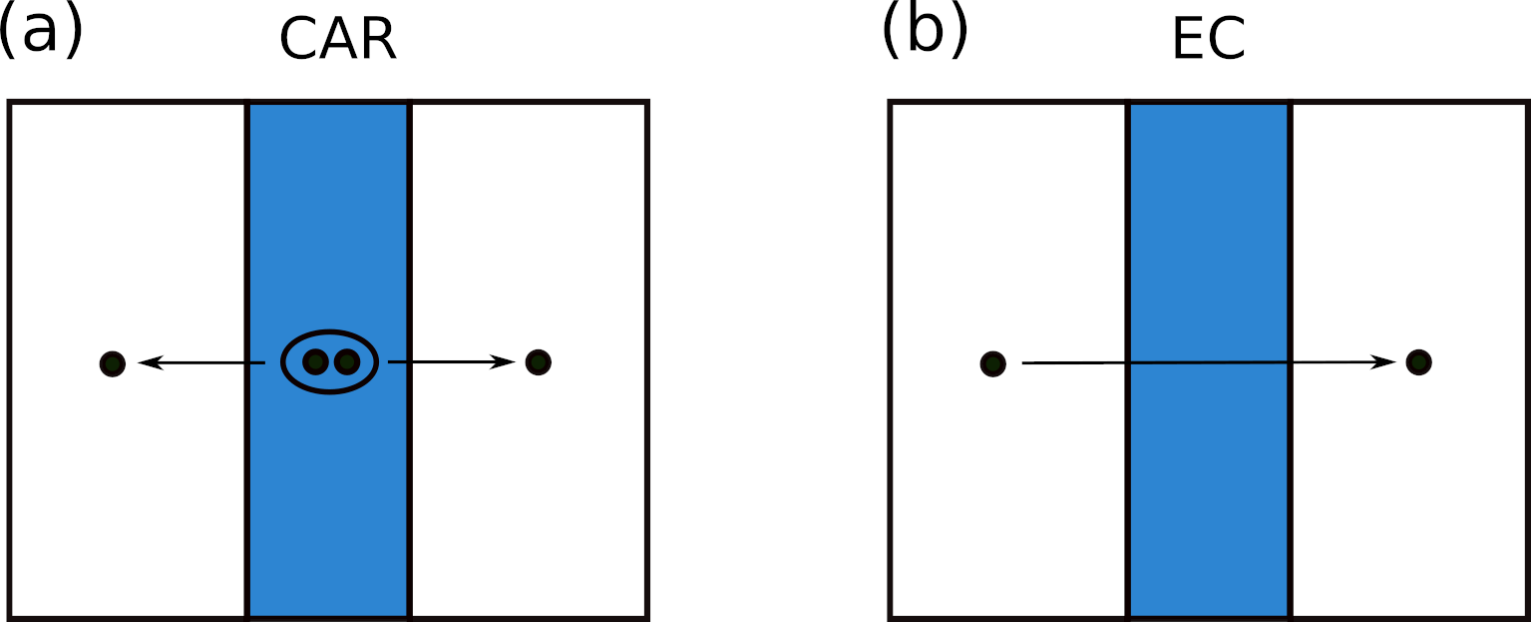
Schematics of the processes of crossed Andreev reflection (a) and elastic cotunneling (b). These schemes are redrawn from [25].

Normal metals connected to a superconductor exhibit a variety of non-trivial phenomena associated with the existence of proximity-induced superconducting correlations spreading over long distances at sufficiently low temperatures [1]. One of these phenomena is the so-called crossed Andreev reflection (CAR): A Cooper pair may split into two electrons [2] (see Figure 1a), thereby generating pairs of entangled electrons in different metallic electrodes [3]. This phenomenon and its effect on electron transport in normal metal–superconductor–normal metal (NSN) hybrid structures were intensively investigated both theoretically [4–10] and experimentally [11–18] over the past decades.

The process competing with CAR is the so-called elastic cotunneling (EC), where an electron is transferred from one normal metal to another across an effective barrier created by the energy gap inside the superconductor, see Figure 1b. Unlike CAR, EC does not produce entangled electrons. In the zero-temperature limit, CAR and EC contributions to the low-bias non-local conductance of an NSN device cancel each other in the limit of low-transparency barriers [4]. In contrast, at high transmissions, the CAR contribution vanishes [6–7]. These observations make an unambiguous identification of CAR in transport experiments a non-trivial task.

The way out is to investigate fluctuations of electric currents passing through both NS boundaries of an NSN structure. While in non-superconducting multiterminal structures cross correlations of current noise in different terminals always remain negative [19], such cross correlations may become positive in the presence of superconductivity due to the process of CAR. This conclusion was initially reached theoretically in the limit of low-transparency barriers at NS interfaces [20–21] and later extended to the case of arbitrary barrier transmissions [22–25]. Positively cross-correlated non-local shot noise was indeed observed in a number of experiments [26–27]. Real-time observation of Cooper pair splitting was also reported in a recent work [28].

Usually, an interplay between positive and negative cross correlations of current noise in NSN devices can be controlled and tuned by applying external bias voltages. In this work we suggest another way of controlling Cooper pair splitting: We predict and investigate non-trivial behavior of cross-correlated non-local shot noise in the presence of a temperature gradient. Note that, previously, this so-called “delta-T” noise was studied in normal atomic-scale junctions [29]. Here, we demonstrate that such kind of noise can also manifest itself in subtle non-local properties of hybrid NSN structures associated with the phenomena of crossed Andreev reflection and Cooper pair splitting.

## Results and Discussion

**Figure 2:**
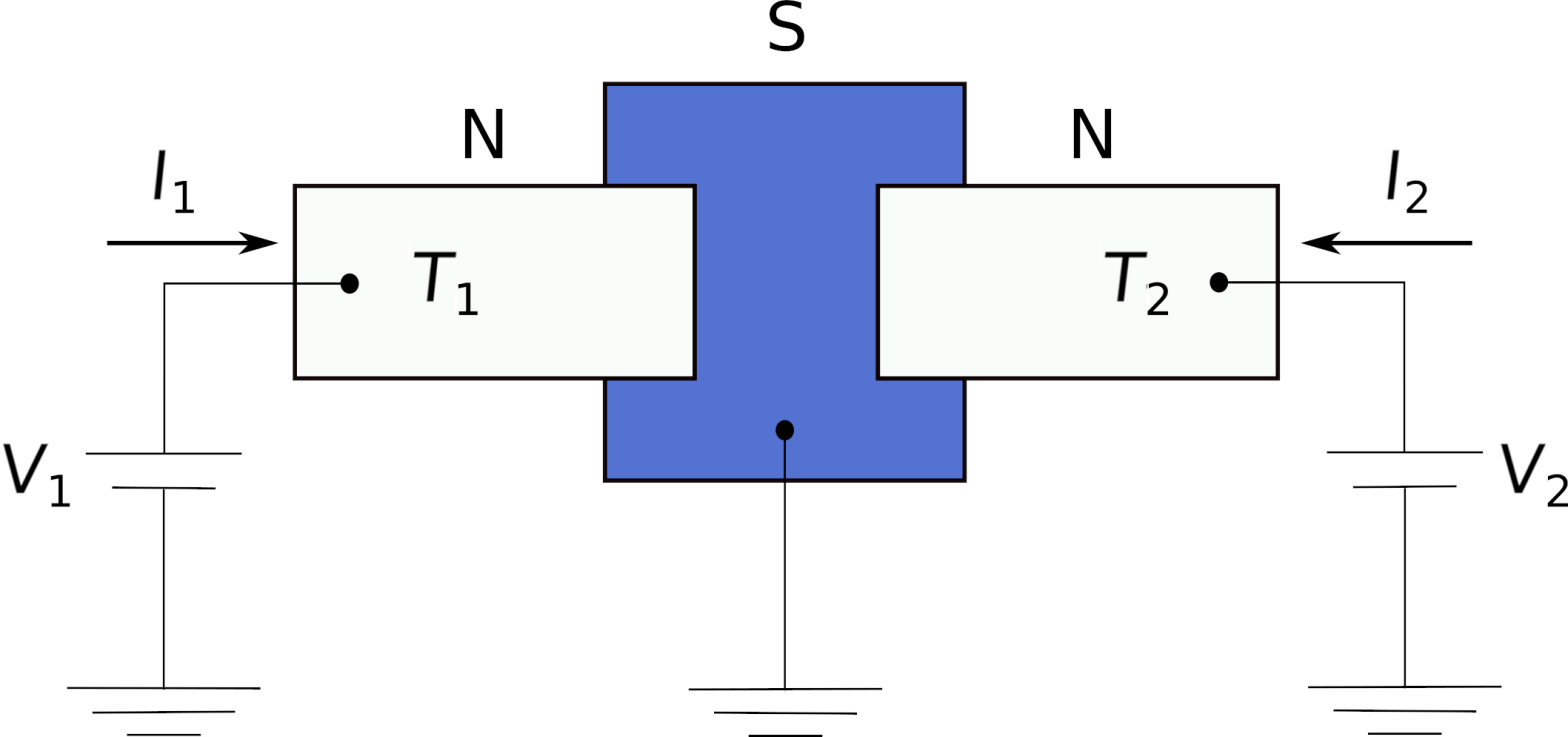
Schematics of the NSN structure under consideration. Normal electrodes are biased by external voltages *V*_1_ and *V*_2_ and are kept at different temperatures *T*_1_ and *T*_2_. The superconducting electrode is assumed to be thinner than the superconducting coherence length ξ.

Let us consider the NSN structure depicted in Figure 2. Normal metallic leads are attached to a bulk superconductor with the aid of two junctions described by a set of conducting channel transmissions τ_1,_*_n_* and τ_2,_*_n_* with *n* being the integer number enumerating all conducting channels. The two junctions are located at a distance considerably shorter than the superconducting coherence length ξ. Normal electrodes are kept at different temperatures *T*_1_ and *T*_2_, thus creating a temperature gradient across our device. In addition, voltages *V*_1_ and *V*_2_ can be applied to two normal leads, as shown in Figure 2.

The Hamiltonian of this structure can be chosen in the form


[1]

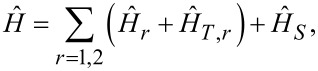



where


[2]

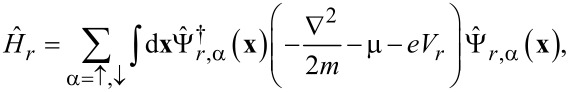



are the Hamiltonians of two normal leads, 
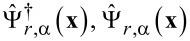
 denote creation and annihilation operators for an electron with a spin projection α at a point **x**, *m* is the electron mass, and μ is the chemical potential,


[3]

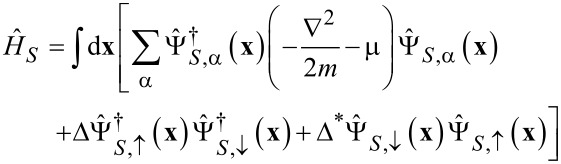



is the Hamiltonian of a superconducting electrode with the order parameter Δ and the terms


[4]

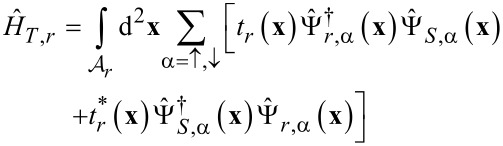



account for electron transfer through the junctions between the superconductor and the normal leads. In Equation 4, the surface integrals are taken over the contact areas 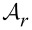, and *t_r_*(**x**) denote coordinate- and spin-independent tunneling amplitudes.

Let us denote the probability for *N*_1_ and *N*_2_ electrons to be transferred, respectively, through the junctions 1 and 2 during the observation time *t* as *P_t_*(*N*_1_,*N*_2_). Introducing the so-called cumulant generating function 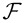
(χ_1_,χ_2_) by means of the formula


[5]

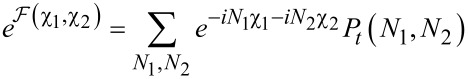



with χ_1,2_ being the counting fields, one can express the average currents through the junctions 
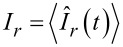
, and the current–current correlation functions


[6]

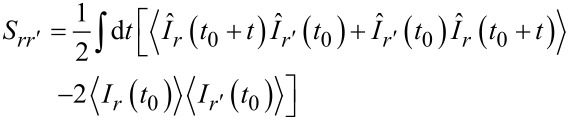



in the following form


[7]

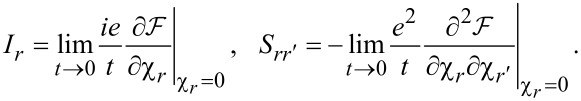



The cumulant generating function 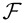 in Equation 5 be evaluated in a general form with the aid of the path integral technique [22,25], which yields


[8]

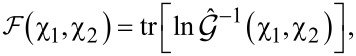



where 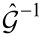 is the Keldysh Green function of our system


[9]

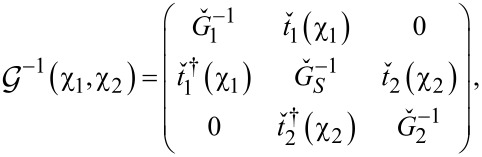



the 4 × 4 matrices 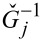 represent the inverse Keldysh Green functions of isolated normal and superconducting leads and 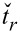 is the diagonal 4 × 4 matrix in the Nambu–Keldysh space describing tunneling between the leads,


[10]

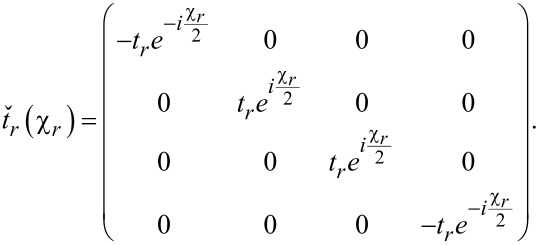



Further analysis of the general expression for the function 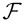 (Equation 8) is essentially identical to that already carried out in [25]. Therefore, it is not necessary to go into details here. Employing Equation 7 and making use of the results [25], we recover general expressions for both the currents *I_r_* across the junctions and the cross-correlated current noise *S*_12_ in the presence of a temperature gradient between two normal terminals.

In what follows we will be particularly interested in the limit of low voltages and temperatures *eV*_1,2_,*T*_1,2_ ≪ Δ. In this case, *I_r_* (containing both local and non-local components) is practically insensitive to temperature and matches with the results [6–7,10] derived earlier in the corresponding limit.

For the non-local current noise in the same limit *eV*_1,2_,*T*_1,2_ ≪ Δ we obtain


[11]

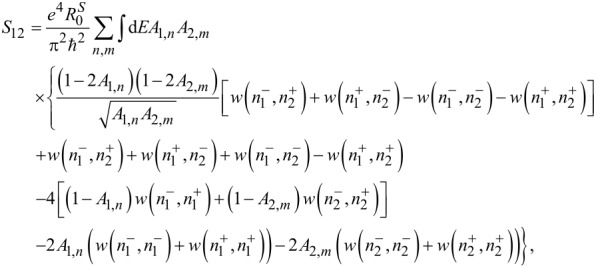



where we defined the Andreev reflection probabilities per conducting channel in both junctions


[12]

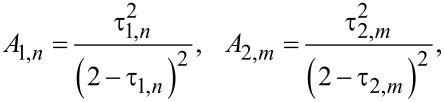



introduced the function


[13]

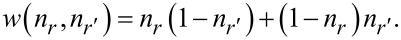



and employed Fermi distribution functions for electrons and holes in the normal leads


[14]

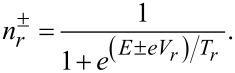



Equation 11 defines the low-energy cross-correlated current noise in the presence of a temperature gradient and represents the main general result of the present work.

The expression (Equation 11) contains the integrals of the type 
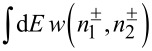
, which cannot be handled analytically except in some special limits, i.e.,


[15]

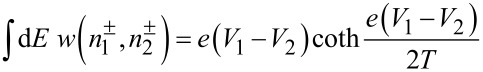



for *T*_1_ = *T*_2_ = *T* and


[16]

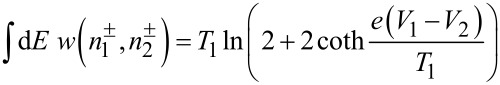



for *T*_1_ ≫ *T*_2_. In the opposite limit *T*_2_ ≫ *T*_1_ in Equation 16 one should simply interchange *T*_1_ ↔ *T*_2_.

In order to proceed, we note that there exists a very accurate interpolation formula between the above limits. It reads


[17]

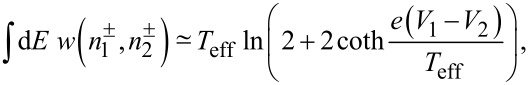



where we defined an effective temperature


[18]

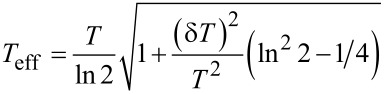



and introduced the notations *T* = (*T*_1_ + *T*_2_)/2 and δ*T* = *T*_1_ − *T*_2_.

With the aid of this interpolation, the non-local noise (Equation 11) can be reduced to a much simpler form


[19]

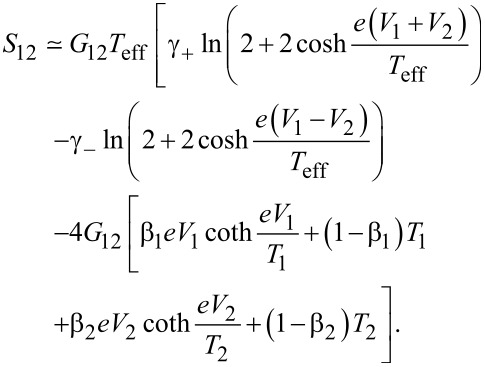



Here we have introduced the non-local conductance in the limit of zero temperature and zero bias voltage,


[20] $$G_{12}=\frac{2e^{4}R_{0}^{S}}{\pi^{2}}\left(\sum_{n}A_{1,n}\right)\left(\sum_{m}A_{2,m}\right),$$

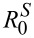
 being the normal state resistance of a superconducting island [25], as well as the parameters


[21]

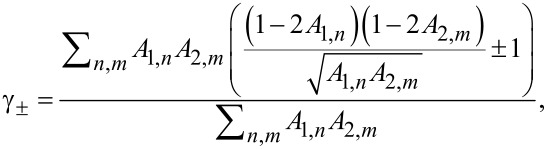



and the local Fano factors for two barriers in the Andreev reflection regime


[22]

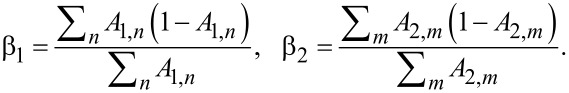



At zero bias voltages *V*_1_ = *V*_2_ = 0, we obtain the noise in the form


[23]

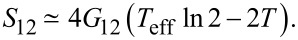



Hence, for the excess non-local noise δ*S*_12_ = *S*_12_(*T*,δ*T*) − *S*_12_(*T*,0) induced by the temperature gradient we get


[24]

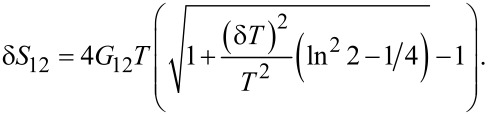



This contribution is positive and reaches its maximum δ*S*_12_ ≃ 0.44*G*_12_*T* at |δ*T*| = *T*.

The effect of the temperature gradient on cross-correlated non-local noise remains appreciable also at non-zero bias voltages *V*_1,2_, in which case it essentially depends on transmission distributions in both junctions.

We start from the tunneling limit *A*_1,_*_n_*, *A*_2,_*_m_* ≪ 1, where one has


[25]

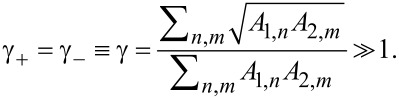



Keeping only the terms ∝γ_±_ in the expression (Equation 19), we obtain


[26]

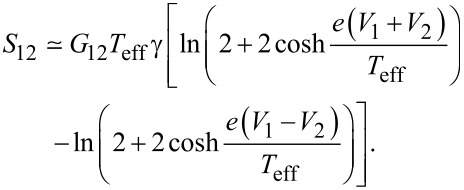



**Figure 3:**
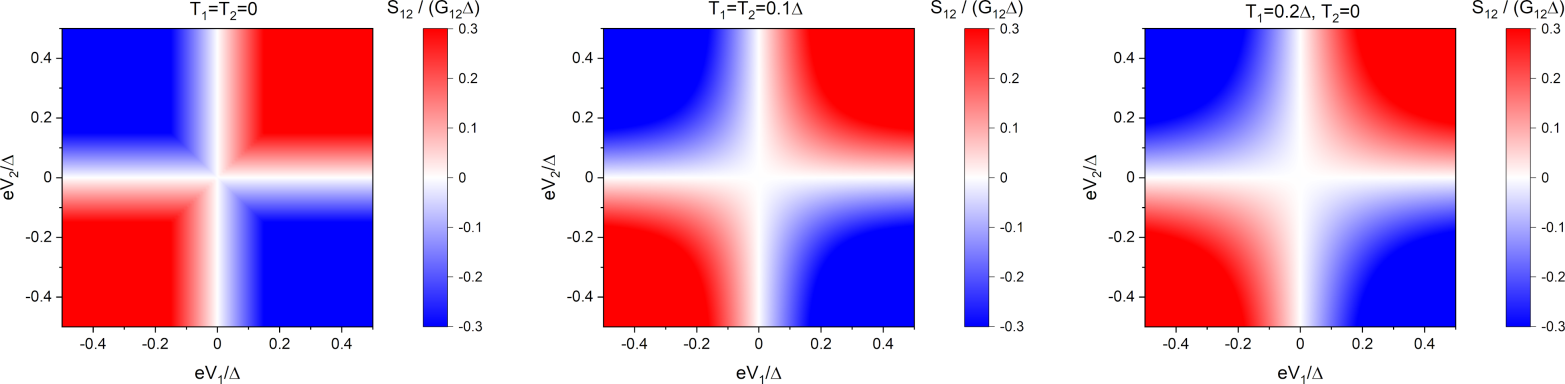
Non-local noise *S*_12_ (Equation 26) in the tunneling limit (Equation 25). Left panel: *T* = 0; middle panel: *T* = 0.1Δ, δ*T* = 0; right panel: |δ*T*| = *T* = 0.1Δ.

The first and the second terms on the right-hand side of this formula are attributed, respectively, to CAR and EC processes. We observe that, similarly to the limit δ*T* = 0, the noise cross correlations remain positive, *S*_12_ *>* 0, provided *V*_1_ and *V*_2_ have the same sign, and they turn negative, *S*_12_ *<* 0, should *V*_1_ and *V*_2_ have different signs. The result is also illustrated in Figure 3.

In the opposite limit of perfectly conducting channels in both junctions with τ_1,_*_n_* = τ_2,_*_m_* = 1 one gets γ_+_ = 2, γ_−_ = 0, β_1_ = β_2_ = 0. Hence, in this case, Equation 19 yields


[27]

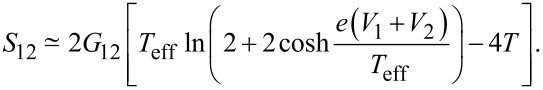



**Figure 4:**
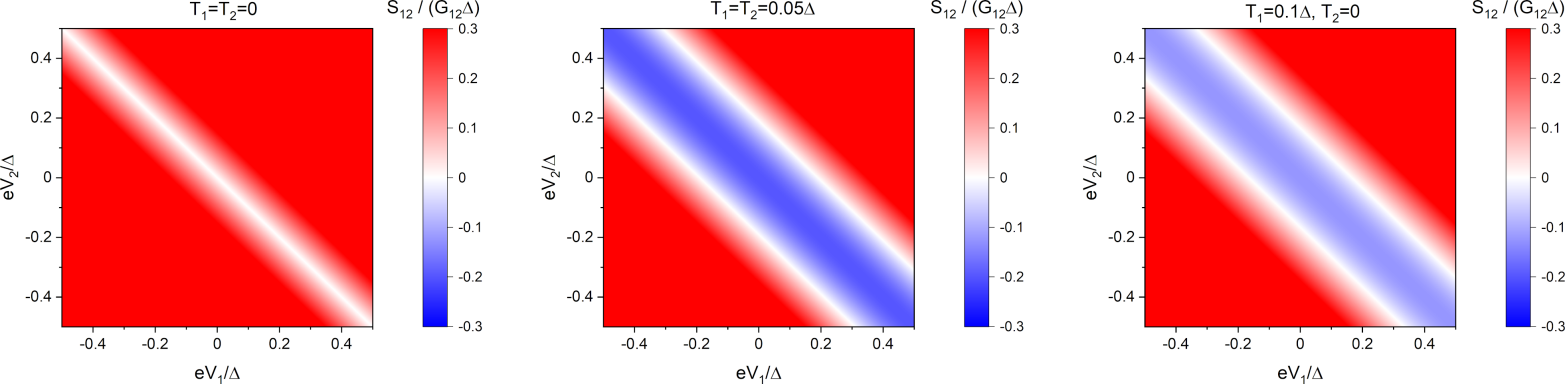
Non-local noise *S*_12_ (Equation 27) in the case of fully transparent junctions. Left panel: *T* = 0; middle panel: *T* = 0.05Δ, δ*T* = 0; right panel: |δ*T*| = *T* = 0.05Δ.

This result is always positive at non-zero bias and sufficiently low temperatures, indicating the importance of CAR processes in this limit, see also Figure 4.

Yet another important physical limit is realized provided the contact has the form of a short diffusive wire with the corresponding Thouless energy exceeding the superconducting gap Δ. In this diffusive limit the transmission probability distributions in both junctions are defined by the well-known formula


[28]

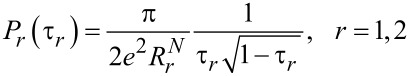



with 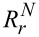 being the resistances of diffusive contacts in the normal state. Making use of this formula, one readily finds γ_±_ = ±1 and β_1_ = β_2_ = 1/3. Then Equation 19 reduces to


[29]

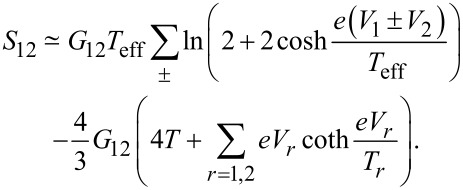



**Figure 5:**
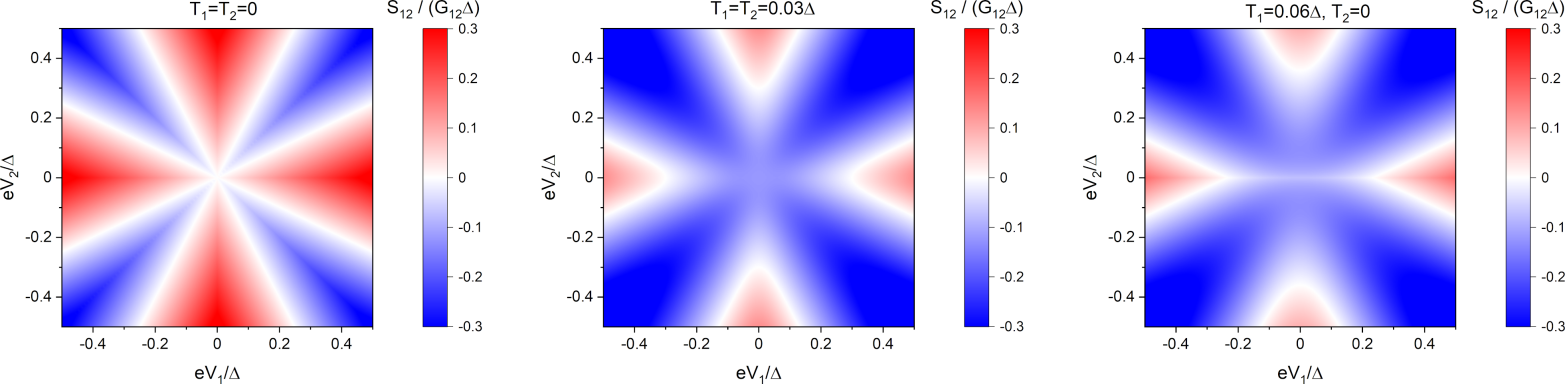
Non-local noise *S*_12_ (Equation 29) in the case of diffusive barriers. Left panel: *T* = 0; middle panel: *T* = 0.03Δ, δ*T* = 0; right panel: |δ*T*| = *T* = 0.03Δ.

This result is also displayed in Figure 5.

## Conclusion

Comparing the non-local shot noise pattern in all the above limits, we can make several important conclusions. First, in full accordance with our previous results [22,25], this pattern turns out to be markedly different depending on particular transmission distributions for intermetallic barriers, thus emphasizing different roles played by CAR and EC processes. Second, relative contributions of the latter processes can be reliably controlled by applied external bias voltages *V*_1_ and *V*_2_ as well as by varying temperature *T*. Third, we observe that the non-local shot noise patterns undergoes additional modifications provided a temperature gradient is applied to our structure. Hence, the temperature gradient, along with other parameters, can also serve as a possible extra tool to control and tune the process of Cooper pair splitting in multiterminal hybrid normal–superconducting metallic structures.
